# Synergy Between *Pseudomonas aeruginosa* Filtrates And Voriconazole Against *Aspergillus fumigatus* Biofilm Is Less for Mucoid Isolates From Persons With Cystic Fibrosis

**DOI:** 10.3389/fcimb.2022.817315

**Published:** 2022-04-14

**Authors:** Gabriele Sass, Julianne J. Marsh, Pallabi Shrestha, Raquel Sabino, David A. Stevens

**Affiliations:** ^1^Infectious Disease Research Laboratory, California Institute for Medical Research, San Jose, CA, United States; ^2^Department of Infectious Diseases, National Institute of Health, ‘Dr. Ricardo Jorge’, Lisbon, Portugal; ^3^Institute of Environmental Health, Faculty of Medicine, University of Lisbon, Lisbon, Portugal; ^4^Division of Infectious Diseases and Geographic Medicine, Department of Medicine, Stanford University School of Medicine, Stanford, CA, United States

**Keywords:** *Pseudomonas aeruginosa*, *Aspergillus fumigatus*, voriconazole, microbial interaction, cystic fibrosis, drug interaction, therapy

## Abstract

Persons with cystic fibrosis (CF) frequently suffer from *Pseudomonas aeruginosa* and *Aspergillus fumigatus* co-infections. There is evidence that co-infections with these interacting pathogens cause airway inflammation and aggravate deterioration of lung function. We recently showed that *P. aeruginosa* laboratory isolates synergistically interact with the anti-fungal azole voriconazole (VCZ), inhibiting biofilm metabolism of several *A. fumigatus* laboratory strains. Interaction was usually mediated *via* pyoverdine, but also *via* pyocyanin or pyochelin. Here we used planktonic filtrates of 7 mucoid and 9 non-mucoid *P. aeruginosa* isolates from CF patients, as well as 8 isolates without CF origin, and found that all of these isolates interacted with VCZ synergistically at their IC50 as well as higher dilutions. CF mucoid isolates showed the weakest interactive effects. Four non-mucoid *P. aeruginosa* CF isolates produced no or very low levels of pyoverdine and did not reach an IC50 against forming *A. fumigatus* biofilm; interaction with VCZ still was synergistic. A VCZ-resistant *A. fumigatus* strain showed the same level of susceptibility for *P. aeruginosa* anti-fungal activity as a VCZ-susceptible reference strain. Filtrates of most *Pseudomonas* isolates were able to increase anti-fungal activity of VCZ on a susceptible *A. fumigatus* strain. This was also possible for the VCZ-resistant strain. In summary these data show that clinical *P. aeruginosa* isolates, at varying degrees, synergistically interact with VCZ, and that pyoverdine is not the only molecule responsible. These data also strengthen the idea that during co-infections of *A. fumigatus* and *P. aeruginosa* lower concentrations of VCZ might be sufficient to control fungal growth.

## Introduction

Co-infections with bacteria and fungi can worsen the course of infection, e.g., in persons suffering from cystic fibrosis (CF). Among such co-infections, combinations of *Aspergillus fumigatus* with *Pseudomonas aeruginosa*, members of the *Burkholderia cepacia* complex, respiratory syncytial virus, and influenza virus are prominent and have severe impact on lung function (King et al., 2016; O'Brien and Fothergill, 2017). *A. fumigatus* and *P. aeruginosa* co-infections trigger more severe outcomes than each mono-infection (Amin et al., 2010; Reece et al., 2017). To control such infections multiple drugs are used, e.g., targeted therapy to correct CFTR (cystic fibrosis transmembrane conductance regulator) deficiency in persons with CF and eliminate mucus accumulation as the basis for severe infections, or drugs targeting individual pathogens. To treat *A. fumigatus* infections, different azoles, e.g., voriconazole (VCZ), itraconazole, or posaconazole are used (Chishimba et al., 2012; Hogan and Denning, 2011). Occasionally, *Aspergillus* develops resistance against azoles, mainly based on mutations in *cyp51A* gene (Arastehfar et al., 2021).

*Pseudomonas* competes with *Aspergillus* for crucial resources, e.g., iron, *via* a variety of their products, such as phenazines, its major siderophore pyoverdine (Sass et al., 2017), pyocyanin (5-N-methyl-1-hydroxyphenazine) (Kerr et al., 1999; Briard et al., 2015; Sass et al., 2020), 1-hydroxyphenazine (Kerr et al., 1999; Briard et al., 2015), phenazine-1-carboxamide (Briard et al., 2015), phenazine-1-carboxylic acid (Briard et al., 2015), and rhamnolipids (Briard et al., 2017; Sass et al., 2021). Using laboratory reference isolates (PA14, PAO1) we recently showed that the *Pseudomonas* products pyoverdine, pyocyanin, and pyochelin interacted synergistically with the anti-fungal drug VCZ against *A. fumigatus* forming biofilm *in vitro* (Sass et al., 2021). As correctly emphasized in a recent excellent review, “Pathogens are known to adapt to the host environment during chronic infections; therefore, testing reference strains alongside clinical isolates is extremely important in polymicrobial communication studies” (Reece et al., 2021). Here we studied the relevance of microbial interaction on drug effects using clinical isolates. Given the heterogenicity of clinical *Pseudomonas* isolates, particularly of those from persons with CF (Ferreira et al., 2015), we compared CF and non-CF isolates.

## Materials and Methods

### Materials

2,3-bis(2-methoxy-4-nitro-5-sulfophenyl)-2H-tetrazolium-5-carboxanilide inner salt (XTT), menadione, rhamnolipids, and RPMI 1640 medium were purchased from Sigma-Aldrich (St. Louis, MO). Iron content in RPMI 1640 medium was below the detection limit (<1 µM, measured by inductively coupled plasma optical emission spectroscopy by Paolo Visca, Rome, Italy, personal communication). Voriconazole (VCZ) was obtained from Pfizer, New York City. Stock was prepared in DMSO and was further diluted to test conditions in RPMI. DMSO concentration in our combination experiments was 0.01%. DMSO concentrations below 1% do not affect *A. fumigatus* biofilm metabolism, thus did not require separate DMSO controls. Large batches of the reagents were prepared in aliquots and frozen, and a fresh aliquot was used in each experiment.

### Strains and Isolates

The use of all microbes in our laboratory is approved by the CIMR Biological Use Committee (approval no. 001-03Yr.16). Assays were performed using the A. *fumigatus* virulent patient isolate 10AF [ATCC 90240 (Denning et al., 1990; Denning and Stevens, 1991)], or a VCZ-resistant isolate (AF21-23) with the most common mutation, TR34/L98H, in the promotor and within the *cyp51A* gene, which encodes the 14-demethylase enzyme critical to ergosterol synthesis (Sabino et al., 2021). Sixteen *P. aeruginosa* isolates from CF patients (7 with mucoid phenotype and 9 with non-mucoid phenotype) and eight isolates of *P. aeruginosa* recovered from non-CF patients were randomly chosen from a library of patient cultures, and obtained after written informed consent, for biobanking of the specimens and subsequent use of the patients, approved by the Stanford University (SU) Institutional Review Board. Another isolate was obtained following clinically indicated cultures from the Santa Clara Valley Medical Hospital (VMC). All isolates are shown in **Table 1**. In CF airways *P. aeruginosa* evolves into variants, such as mucoid colony types, which are adapted to chronic residence there (Davis et al., 1967; Davies and Bilton, 2009; Folkesson et al., 2012; Ferreira et al., 2015). For comparison, the *P. aeruginosa* laboratory reference isolates PA14 (O'Toole and Kolter, 1998; Lee et al., 2006; Fischer et al., 2016) and PAO1 (ATCC 15692) (Stover et al., 2000) were used.

**Table 1:** Clinical *P. aeruginosa* isolates used in this study.

CIMR#	Clinical Laboratory ID # Santa Clara Valley Medical Center (VMC) or Stanford University (SU)	Specimen	Phenotype
14-79	SU20060455	Respiratory	CF mucoid
14-92	SU09710807	Respiratory	CF mucoid
14-97	SU16242976	Respiratory	CF mucoid
14-112	SU40721045	Respiratory	CF mucoid
14-115	SU40943938	Respiratory	CF mucoid
14-118	SU08215535	Respiratory	CF mucoid
14-122	SU60141132	Respiratory	CF mucoid
14-81	SU09710807	Respiratory	CF non-mucoid
14-82	SU16242976	Respiratory	CF non-mucoid
14-84	SU7841943	Respiratory	CF non-mucoid
14-89	SU41053570	Respiratory	CF non-mucoid
14-113	SU23373137	Respiratory	CF non-mucoid
14-114	SU23373137	Respiratory	CF non-mucoid
14-116	SU40943938	Respiratory	CF non-mucoid
14-117	SU60908696	Respiratory	CF non-mucoid
14-119	SU06034359	Respiratory	CF non-mucoid
14-75	VMC	Respiratory	Non-CF
14-78	SU60370871	Respiratory	Non-CF
14-86	SU42266353	Respiratory	Non-CF
14-90	SU27917939	Respiratory	Non-CF
14-91	SU27917939	Respiratory	Non-CF
14-93	SU21548292	Respiratory	Non-CF
14-98	SU41082579	Non-Respiratory	Non-CF
14-101	SU28674323	Non-Respiratory	Non-CF

### *P. aeruginosa* Planktonic Filtrate Production and Pyoverdine Measurement

*P. aeruginosa* filtrates were prepared as detailed previously (Ferreira et al., 2015). Briefly, *P. aeruginosa* bacteria (5 × 10^7^ cells/ml) were incubated in RPMI 1640 medium (Sigma-Aldrich) at 37°C and 100 rpm for 24 h. Bacterial growth was measured at 600 nm using a spectrophotometer (Genesys 20, Thermo Fisher Scientific Inc., Waltham, MA). Bacterial cultures were centrifuged at 200×*g* for 30 min at room temperature, and filtered for sterility (0.22 μm). Pyoverdine production was measured at 405 nm. Measurements were normalized to bacterial growth using the formula: Relative pyoverdine production = OD 405/OD600, forming a pyoverdine quotient (Sass et al., 2017).

### Assay for Measurement of *Aspergillus* Forming Biofilm Metabolism

*A. fumigatus* conidia (10^5^/ml final concentration) were distributed into the wells of sterile flat-bottom 96-well culture plates at 50 µl/well. Bacterial supernatants or test substances and VCZ were combined in equal parts by volume (25 μl each) to the final concentrations indicated. Final volumes in wells during assays were 100 µl. RPMI 1640 medium served as the negative control. The assay plates were incubated at 37°C overnight and hyphae growth was verified by optical microscopy before performing XTT assays.

All experiments were evaluated by XTT metabolic assay as detailed previously (Scudiero et al., 1988; Ferreira et al., 2015). Briefly, 150 µl of an XTT/menadione mixture (150 µg/ml XTT, 30 µM menadione) were added to each test well and incubated at 37°C for 1 h. Supernatants from each well were transfered to a fresh 96-well plate (100 µl), and assayed using a plate reader (Vmax, Molecular Devices, San Jose, CA) at 490 nm.

### Determination of the Isolate Dilution With 50% Anti-Fungal Activity (IC50)

Filtrates were diluted in RPMI in 1:2 steps with final concentrations ranging from 1:2 to 1:1,024. The concentration closest to inhibiting 50% of fungal metabolism here is referred to as the IC50 of an isolate.

### BLISS Independence Model for Analysis of Drug Combination Effects

Combined drug effects were calculated using the BLISS Independence Model as described previously (Zhao et al., 2014) when combining drugs at their IC50, the optimal concentration for reagent interactions. Briefly, if drugs A (VCZ) and B (*P. aeruginosa* supernatant) inhibit Y_a_ and Y_b_ % of growth, respectively, their predicted combined effect (considering they work independently) is given by the formula: ${\text{Y}}_{\text{ab}}^{\text{p}}$ **= Y_a_ + Y_b_ − Y_a_Y_b_**. The predicted combined effect was compared to the observed combined effect (Y°_ab_, anti-fungal activity by the drug combination in XTT assays). Results were interpreted as:

Observed > Predicted: Synergy (S)
Observed = Predicted: Independent (5% range of ${\text{Y}}_{\text{ab}}^{\text{p}}$ ) (I)
Observed < Predicted: Antagonism (A)

(Abbreviations: Y_a_ = inhibition of fungal metabolism by VCZ, Y_b_ = inhibition of fungal metabolism by Pa sup, Y°_ab_ = observed combined antifungal effect, ${\text{Y}}_{\text{ab}}^{\text{p}}$ = predicted combined antifungal effect).

### Statistical Analysis

Results were analyzed using Student’s *t* test, if two groups were compared, and 1-way ANOVA combined with a Tukey’s post-test for multiple comparisons. All data in this study are expressed as a mean ± SD. Data are reported as the percent of control. Each assay was performed with three to four biological and technical replicates. Representative experiments are shown.

## Results

### Determination of IC50s for Filtrates of Clinical *P. aeruginosa* Isolates Against *A. fumigatus* 10AF Biofilm Formation

**Figure 1:**
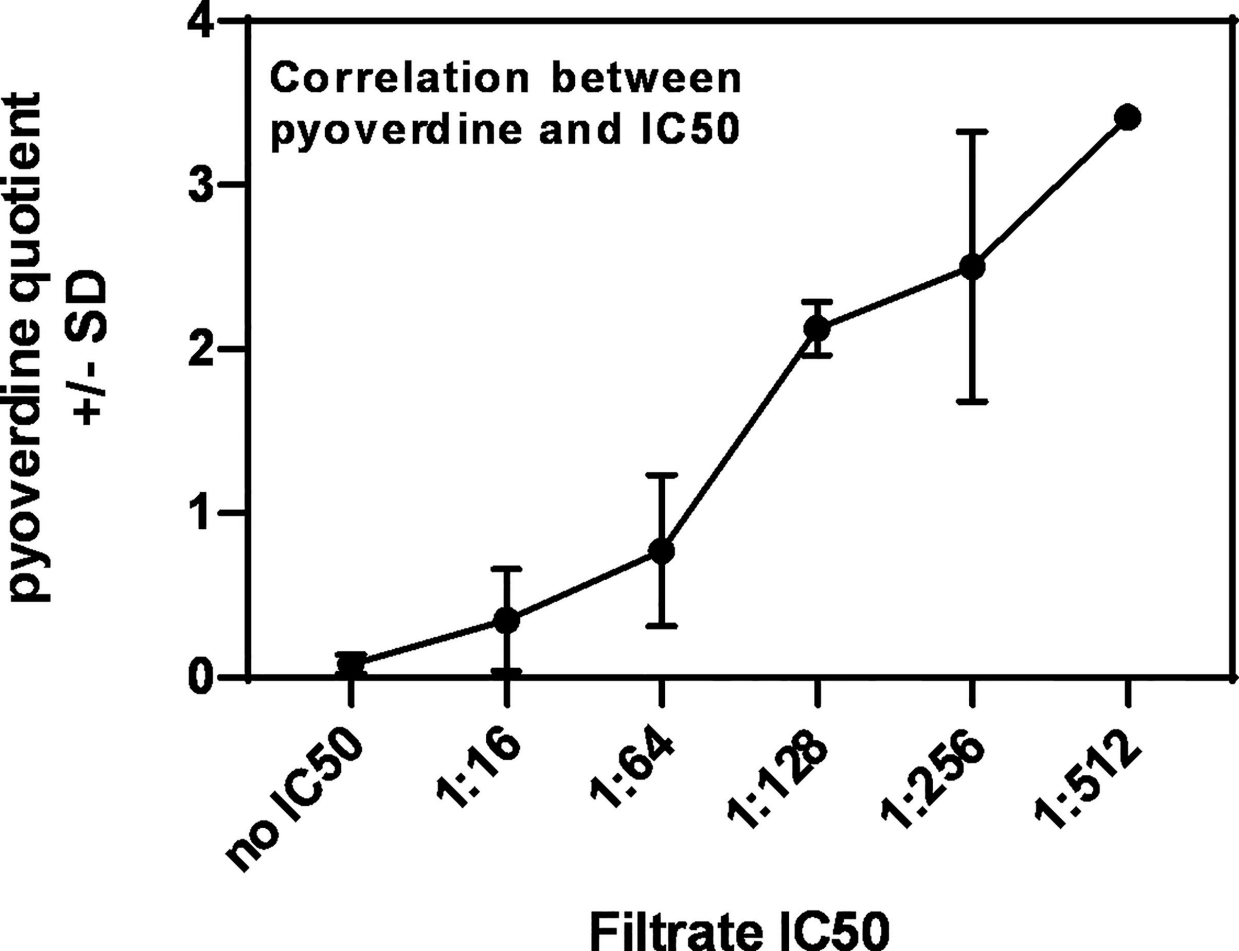
Correlation between pyoverdine content in *Pseudomonas* filtrates, and their IC50s. Pyoverdine quotients shown in **Table 2** were plotted against the individual filtrate’s IC50, revealing a correlation between high pyoverdine content and strong anti-fungal activity.

Planktonic filtrates of seven randomly chosen mucoid or nine non-mucoid clinical *P. aeruginosa* isolates, derived from persons with CF (CF mucoid, CF non-mucoid), or from eight non-CF patients (described in **Table 1**) were produced in RPMI medium under iron-limiting conditions (Sass et al., 2017). Isolate filtrates were tested for anti-fungal activity against 10AF biofilm metabolism in 1:2 dilution steps from 1:2 to 1:1,024. Dose–response curves allowed the determination of dilutions that were within a 2-fold step of the individual IC50 (concentration of agent inhibiting 50%, compared to control). **Table 2** shows that for most isolates an IC50 could be determined, with the dilution closest to the IC50 for individual isolates being between 1:16 and 1:256. Four isolates did not reach an IC50 at any dilution (**Table 2**: isolates 14-82, 14-89, 14-116, and 14-119). The anti-fungal strength of isolates, as assessed by the dilution of filtrate approximating the IC50, closely correlated with their pyoverdine content (**Table 2** and **Figure 1**). These results are in agreement with previous findings (Sass et al., 2017). **Table 2** also shows that mean pyoverdine production and IC50 in the CF mucoid group were lower than observed for the CF non-mucoid or non-CF groups, or for laboratory isolate PA14.

**Table 2:** IC50, BLISS score and pyoverdine content determination for isolates used in this study.

CIMR#	Filtrate dilution closest to IC50	Interaction filtrate IC50/VCZ IC50 BLISS score	Pyoverdine quotient
**CF mucoid**			
14-79	1:64	S	0.90
14-92	1:16	S	0.57
14-97	1:256	S	1.73
14-112	1:256	S	3.78
14-115	1:64	S	0.72
14-118	1:64	S	1.29
14-122	1:256	S	1.72
**CF mucoid group mean**	**1:128**	**S**	**1.53**
**CF non-mucoid**			
14-81	1:256	S	2.83
14-82	No IC50	–	0.06
14-84	1: 128	S	2.01
14-89	No IC50	–	0.04
14-113	1:64	S	0.18
14-114	1:512	S	3.41
14-116	No IC50	–	0.06
14-117	1:256	S	3.97
14-119	No IC50	–	0.17
**CF non-mucoid group means**	**1:256**	**S**	**2.48**
	**no IC50**	**-**	**0.08**
**Non-CF**			
14-75	1:256	S	1.99
14-78	1:256	S	2.80
14-86	1:128	S	2.24
14-90	1:256	S	1.82
14-91	1:256	S	2.48
14-93	1:256	S	2.78
14-98	1:16	S	0.13
14-101	1:256	S	1.63
**Non-CF group mean**	**1:256**	**S**	**1.98**
**PA14**	**1:256**	**S**	**2.23**
**PAO1**	**1:512**	**S**	**3.23**

BLISS score: S: synergy. As indicated in the Table the bold values are 'group means'.

### Synergy Between VCZ and Clinical *P. aeruginosa* Isolates Against *A. fumigatus* 10AF Biofilm Formation

**Figure 2:**
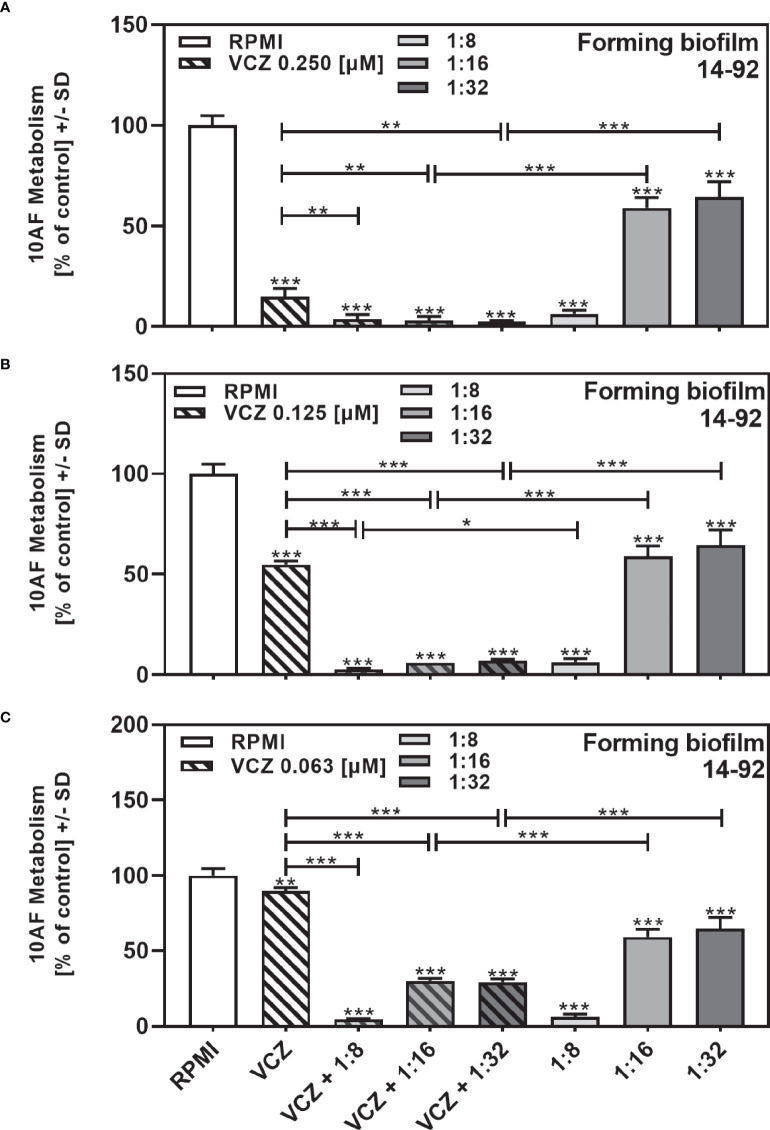
Examples for synergistic anti-fungal effects of the combination of *P. aeruginosa* clinical isolate filtrate and VCZ. Isolate 14-92 filtrate was diluted to final concentrations of 1:8 to 1:32, encompassing its inhibitory concentration of 50% (IC50), and combined with VCZ at 0.25 µM **(A)**, 0.125 µM **(B)**, or 0.063 µM **(C)** to test their combined antifungal activities against 10AF forming biofilm (10^5^ conidia/ml in RPMI 1640 medium). Assay plates were incubated at 37°C overnight. 10AF fungal metabolism was measured by XTT assay. Metabolism in the presence of RPMI alone (white bar) was regarded as 100%. Statistical analysis: Unpaired t-test for VCZ (white striped bar), or each filtrate dilution (solid gray bars), vs. combinations of VCZ and filtrate (striped gray bars): one, two or three asterisks = p ≤0.05, p ≤0.01 or p ≤0.001, respectively.

In a previous study we determined the IC50 for VCZ against 10AF forming biofilm metabolism within a 2-fold dilution step of 125 nM (Sass et al., 2021). We now combined filtrates of each clinical isolate at the dilution closest to its individual IC50 (**Table 2**) with VCZ at its IC50. Combination experiments were performed for all isolates with the exception of the four isolates that did not reach an IC50 (**Table 2**: isolates 14-82, 14-89, 14-116, and 14-119). **Figure 2** gives an example of an isolate (14–92), tested for interaction with VCZ at concentrations surrounding its own IC50 (1:8 to 1:32), in combination with VCZ (**Figure 2A**: 0.25 µM, **Figure 2B**: 0.125 µM, **Figure 2C**: 0.063 µM). Our results show that synergistic effects were achieved, unless anti-fungal activity of one of the components alone was very strong (**Figure 2**).

**Figure 3:**
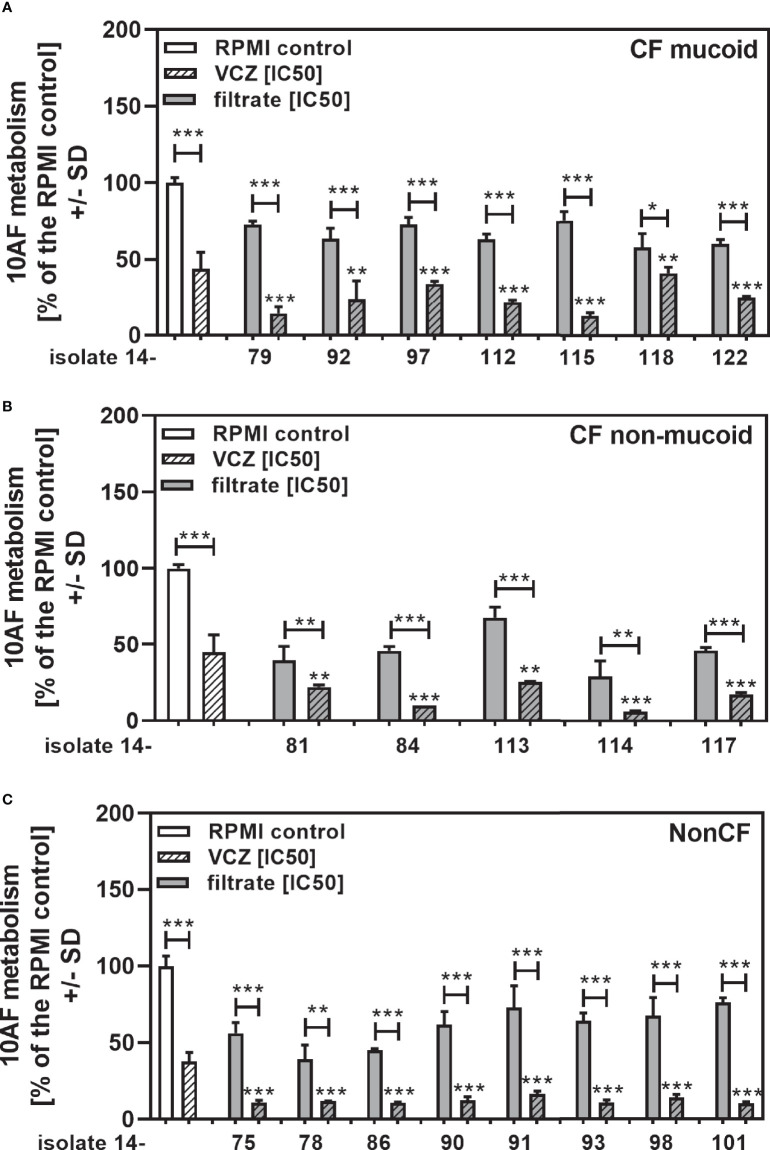
Anti-fungal effects of the combination of CF mucoid, CF non-mucoid, or non-CF *P. aeruginosa* clinical isolate filtrates and VCZ at their IC50s. CF mucoid **(A)**, CF non-mucoid **(B)**, or non-CF isolate filtrates **(C)** were diluted to be used at final concentrations closest to their IC50, and combined with VCZ close to its IC50, to test their combined antifungal activities against 10AF forming biofilm (10^5^ conidia/ml in RPMI 1640 medium). Assay plates were incubated at 37°C overnight. 10AF fungal metabolism was measured by XTT assay. Metabolism in the presence of RPMI alone (white bar) was regarded as 100%. X-axis shows isolate numbers. Comparisons without brackets: VCZ alone (striped white bar) vs. the individual isolate filtrate combination with VCZ (gray striped bar). Other comparisons as indicated by the ends of the brackets. Statistical analysis: Unpaired t-test: one, two or three asterisks = p ≤0.05, p ≤0.01 or p ≤0.001, respectively.

We show each clinical isolate filtrate as well as PA14 filtrate at concentrations closest to their individual IC50s (**Table 2**), combined with VCZ at its IC50 (**Figure 3**). This shows that all individual filtrates had significantly stronger anti-fungal activity when combined with VCZ, independently of the phenotype or origin of the isolates; mucoid (**Figure 3A**), non-mucoid CF (**Figure 3B**), or non-CF (**Figure 3C**). Using the BLISS Independence Model we calculated the type of interaction (synergistic (S), independent (I) or antagonistic (A)) for all filtrate/VCZ combinations. **Table 2** summarizes that all interactions were synergistic.

**Figure 4:**
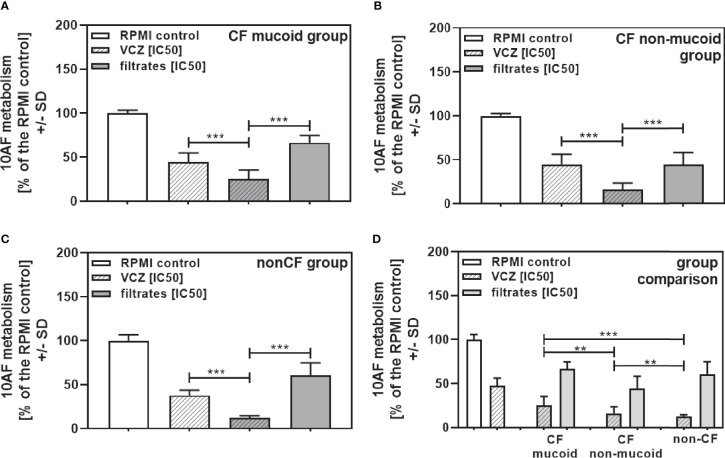
Group comparisons for anti-fungal effects of the combination of CF mucoid, CF non-mucoid, or non-CF *P. aeruginosa* clinical isolate filtrates and VCZ at their IC50s. Individual isolate interactions with VCZ shown in **Figure 3** were combined per group [CF mucoid **(A)**, CF non-mucoid **(B)**, or non-CF isolate filtrates **(C)**]. **(D)** Comparison among effects of VCZ/filtrate combinations, shown in **(A–C)**, on 10AF biofilm formation. Metabolism in the presence of RPMI alone (white bar) was regarded as 100%. Comparisons as indicated by the ends of the brackets. Statistical analysis: Unpaired t-test: two or three asterisks = p ≤0.01 or p ≤0.001, respectively.

When all isolates per group were combined, combinations with VCZ were synergistic as well (**Figure 4A**: mucoid CF, **Figure 4B**: non-mucoid CF, **Figure 4C**: non-CF; **Table 2**). When comparing mucoid CF, non-mucoid CF and non-CF isolates, synergy of mucoid CF isolates with VCZ was found weaker than synergy between VCZ and non-mucoid CF or non-CF isolates (**Figure 4D**). Synergy between isolate filtrates and VCZ was strongest in the non-CF group (**Figure 4D**).

### High Dilutions of Clinical Isolate Filtrates Still Support Anti-Fungal Activity of VCZ

**Figure 5:**
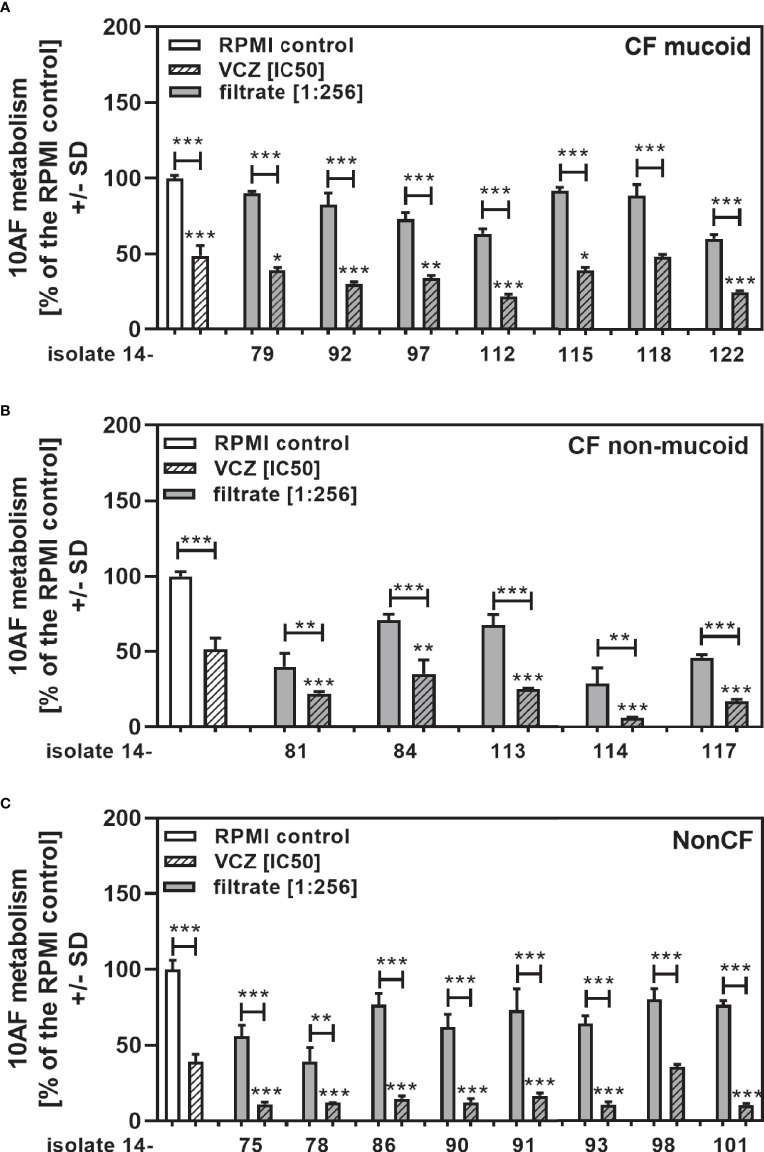
Anti-fungal effects of the combination of CF mucoid, CF non-mucoid, or non-CF *P. aeruginosa* clinical isolate filtrates at 1:256 dilutions with VCZ at its IC50. CF mucoid **(A)**, CF non-mucoid **(B)**, or non-CF isolate filtrates **(C)** were diluted to be studied at final concentrations of 1:256, and combined with VCZ close to its IC50, to test their combined antifungal activities against 10AF forming biofilm (10^5^ conidia/ml in RPMI 1640 medium). Assay plates were incubated at 37°C overnight. 10AF fungal metabolism was measured by XTT assay. Metabolism in the presence of RPMI alone (white bar) was regarded as 100%. X-axis shows isolate numbers. Comparisons without brackets: VCZ alone (striped white bar) vs the individual isolate filtrate combination with VCZ (gray striped bar). Other comparisons as indicated by the ends of the brackets. Statistical analysis: Unpaired t-test: one, two or three asterisks = p ≤0.05, p ≤0.01 or p ≤0.001, respectively.

**Figure 6:**
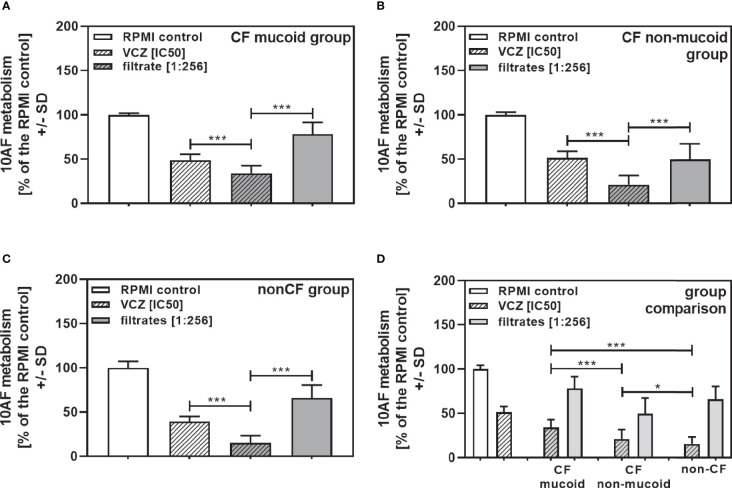
Group comparisons for anti-fungal effects of the combination of CF mucoid, CF non-mucoid, or non-CF *P. aeruginosa* clinical isolate filtrates at 1:256 dilutions with VCZ at its IC50. Individual isolate interactions with VCZ shown in **Figure 4** were combined per group [CF mucoid **(A)**, CF non-mucoid **(B)**, or non-CF isolate filtrates **(C)**]. **(D)** Comparison among effects of VCZ/filtrate combinations, shown in **(A-C)**, on 10AF biofilm formation. Metabolism in the presence of RPMI alone (white bar) was regarded as 100%. Comparisons as indicated by the ends of the brackets. Statistical analysis: Unpaired t-test: one or three asterisks = p ≤0.05 or p ≤0.001, respectively.

To determine how potent interactions of isolates of each group (CF mucoid, CF non-mucoid, non-CF) were with VCZ, we further combined each isolate at the same high filtrate dilution (1:256), with VCZ at its IC50. This also enabled comparisons of the data from studying clinical isolates to those previously published with reference laboratory isolates (Sass et al., 2021), as the latter study investigated filtrates at a 1:256 dilution. **Figure 5** shows that in each group (**Figure 5A**: mucoid CF, **Figure 5B**: non-mucoid CF, **Figure 5C**: non-CF) all isolates acted synergistically with VCZ when used at a 1:256 dilution. The same result was obtained when all isolates per group were combined (**Figure 6A**: mucoid CF, **Figure 6B**: non-mucoid CF, **Figure 6C**: non-CF). Again, combinations of CF mucoid filtrates showed significantly less synergy with VCZ than non-mucoid filtrates or non-CF filtrates, and synergy of non-CF isolates with VCZ was strongest (**Figure 6D**).

### Isolates Not Showing an IC50 Still Support VCZ Anti-Fungal Activity

**Figure 7:**
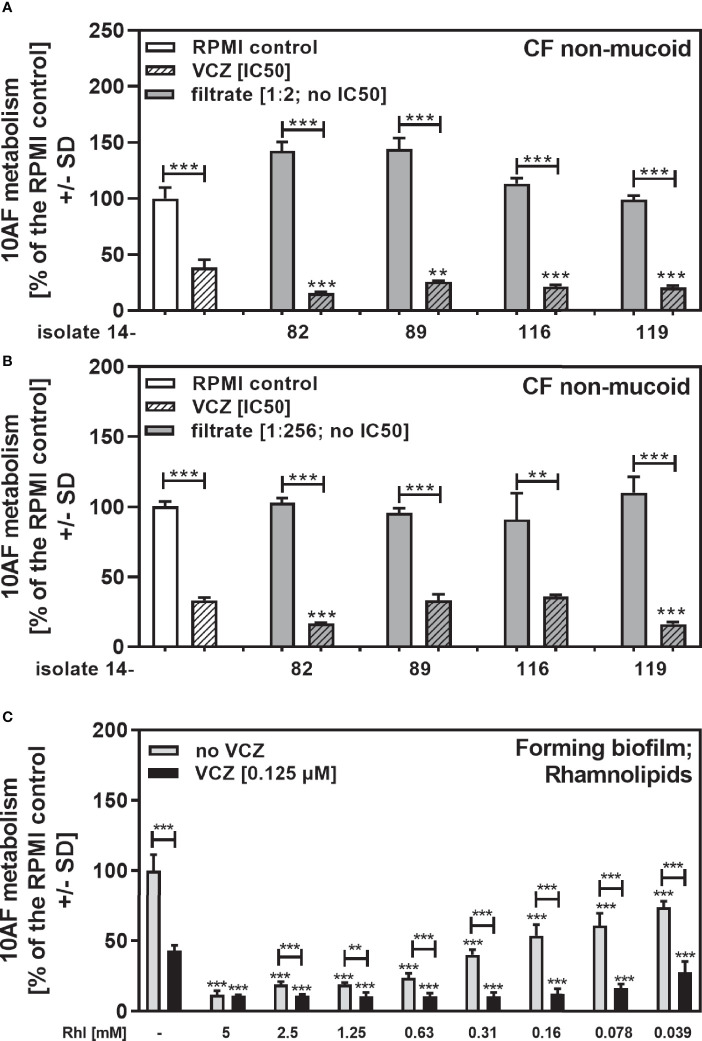
Anti-fungal effects of the combination of pyoverdine-negative *P. aeruginosa* clinical isolate filtrates with VCZ. Four pyoverdine-negative CF non-mucoid isolate filtrates (no IC50) were diluted 1:2 **(A)** or 1:256 **(B)**, and combined with VCZ close to its IC50, to test their combined antifungal activities against 10AF forming biofilm (10^5^ conidia/ml in RPMI 1640 medium). Assay plates were incubated at 37°C overnight. 10AF fungal metabolism was measured by XTT assay. Metabolism in the presence of RPMI alone (white bar) was regarded as 100%. X-axis shows isolate numbers. Comparisons without brackets: VCZ alone (striped white bar) vs. the individual isolate filtrate combination with VCZ (gray striped bar). Other comparisons as indicated by the ends of the brackets. Statistical analysis: Unpaired t-test: two or three asterisks = p ≤0.01 or p ≤0.001, respectively. **(C)** Rhamnolipids were diluted to final concentrations of 5 to 0.039 mM, and tested against 10AF forming biofilm metabolism (10^5^ conidia/ml in RPMI 1640 medium) either alone (gray bars) or in combination with VCZ close to its IC50. Assay plates were incubated at 37°C overnight. 10AF fungal metabolism was measured by XTT assay. Metabolism in the presence of RPMI alone (leftmost bar) was regarded as 100%. Comparisons: RPMI vs all rhamnolipid concentrations, or VCZ (leftmost black bar) vs. all rhamnolipid/VCZ combinations. Other comparisons as indicated by the ends of the brackets. Statistical analysis: 1-way ANOVA for dose-response-curves, unpaired t-test for comparisons indicated by brackets: two or three asterisks = p ≤0.01 or p ≤0.001, respectively.

In the non-mucoid CF group we found four isolates that did not produce pyoverdine, a major *Pseudomonas* anti-fungal factor (Sass et al., 2017), and did not reach an IC50 (14-82, 14-89, 14-116, 14-119 in **Table 2**). This is a phenomenon not previously encountered, in studying wildtype laboratory isolates. We combined 1:2 (**Figure 7A**) or 1:256 dilutions of these filtrates (**Figure 7B**) with VCZ close to its IC50, and found that all 1:2 diluted filtrates interacted synergistically with VCZ (**Figure 7A**). When filtrates were diluted to 1:256 final concentration, two of the isolates still interacted with VCZ synergistically (**Figure 7B**: 14-82 and 14-119), whereas two isolates no longer did (**Figure 7B**: 14-89 and 14-116). These data suggest that pyoverdine is not solely responsible for combined anti-fungal activity of filtrates and VCZ, and that different amounts of one or more other factors are present in filtrates that interact with VCZ. We studied rhamnolipid production (Sass et al., 2021), and found that isolates 14-82 and 14-119 were capable of producing rhamnolipids, whereas isolates 14-89 and 14-116 were not. This ability to produce rhamnolipids correlated with their abilities to interact synergistically with VCZ at high dilutions. We therefore tested effects of rhamnolipids on 10AF forming biofilm metabolism, and also synergy with VCZ near its IC50, and found the IC50 for anti-fungal activity of rhamnolipids was about 160 µM, and synergy with VCZ at rhamnolipid concentrations below 39 µM, the lowest concentration tested (**Figure 7C**).

### Clinical *Pseudomonas* Isolate Filtrates Boost Anti-Fungal Activity of VCZ at Sub-Optimal Concentrations of VCZ, or in VCZ-Resistant *Aspergillus*

**Figure 8:**
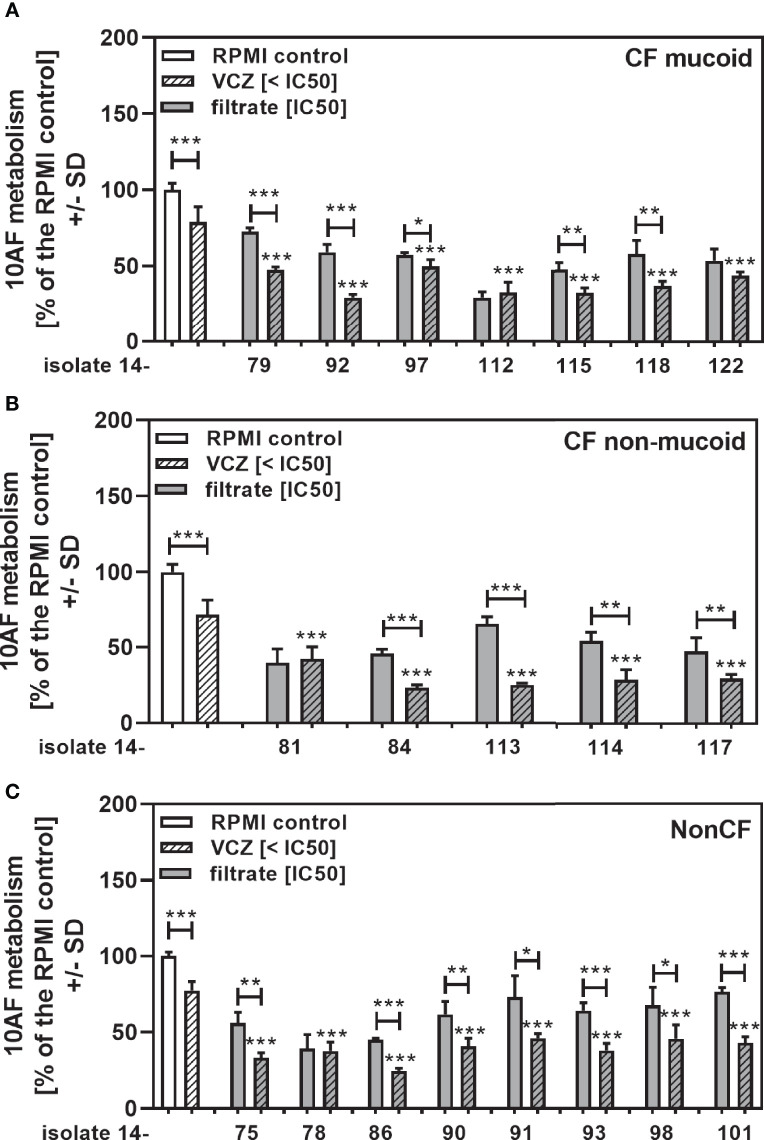
Anti-fungal effects of the combination of CF mucoid, CF non-mucoid, or non-CF *P. aeruginosa* clinical isolate filtrates at their IC50s with VCZ below its IC50. CF mucoid **(A)**, CF non-mucoid **(B)**, or non-CF isolate filtrates **(C)** were diluted to be used at final concentrations closest to their IC50, and combined with VCZ at concentrations below its IC50, to test their combined antifungal activities against 10AF forming biofilm (10^5^ conidia/ml in RPMI 1640 medium). Assay plates were incubated at 37°C overnight. 10AF fungal metabolism was measured by XTT assay. Metabolism in the presence of RPMI alone (white bar) was regarded as 100%. X-axis shows isolate numbers. Comparisons without brackets: VCZ alone (striped white bar) vs. the individual isolate filtrate combination with VCZ (gray striped bar). Other comparisons as indicated by the ends of the brackets. Statistical analysis: Unpaired t-test: one, two or three asterisks = p ≤0.05, p ≤0.01 or p ≤0.001, respectively.

**Figure 9:**
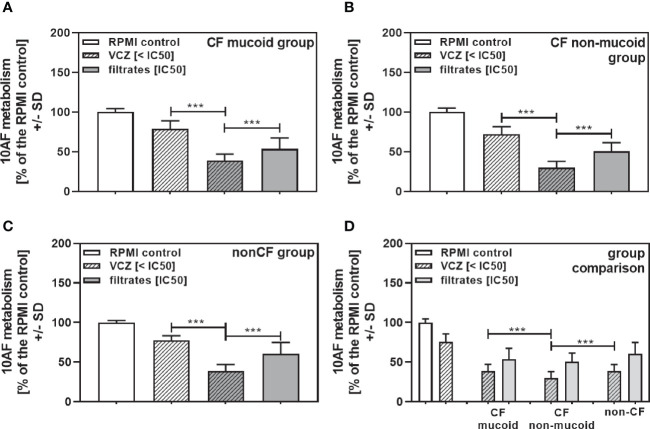
Group comparisons for anti-fungal effects of the combination of CF mucoid, CF non-mucoid, or non-CF *P. aeruginosa* clinical isolate filtrates at their IC50s with VCZ below its IC50. Individual isolate interactions with VCZ shown in **Figure 7** were combined per group [CF mucoid **(A)**, CF non-mucoid **(B)**, or non-CF isolate filtrates **(C)**]. **(D)** Comparison among effects of VCZ/filtrate combinations, shown in **(A–C)**, on 10AF biofilm formation. Metabolism in the presence of RPMI alone (white bar) was regarded as 100%. Comparisons as indicated by the ends of the brackets. Statistical analysis: Unpaired t-test: three asterisks = p ≤0.001.

When clinical isolate filtrates close to their individual IC50s were combined with VCZ at VCZ concentrations that on their own allowed about 25% of anti-fungal activity, we still found synergistic interaction for most of the tested isolates (**Figure 8A**: mucoid CF, **Figure 8B**: non-mucoid CF, **Figure 8C**: non-CF). When all isolates in each group were combined, interactions were synergistic, compared to the single agents (**Figure 9A**: mucoid CF, **Figure 9B**: non-mucoid CF, **Figure 9C**: non-CF). CF mucoid filtrates showed significantly less synergy with VCZ than non-mucoid filtrates, but not less than non-CF isolates, whereas interactions with non-mucoid CF isolates were strongest (**Figure 9D**).

**Figure 10:**
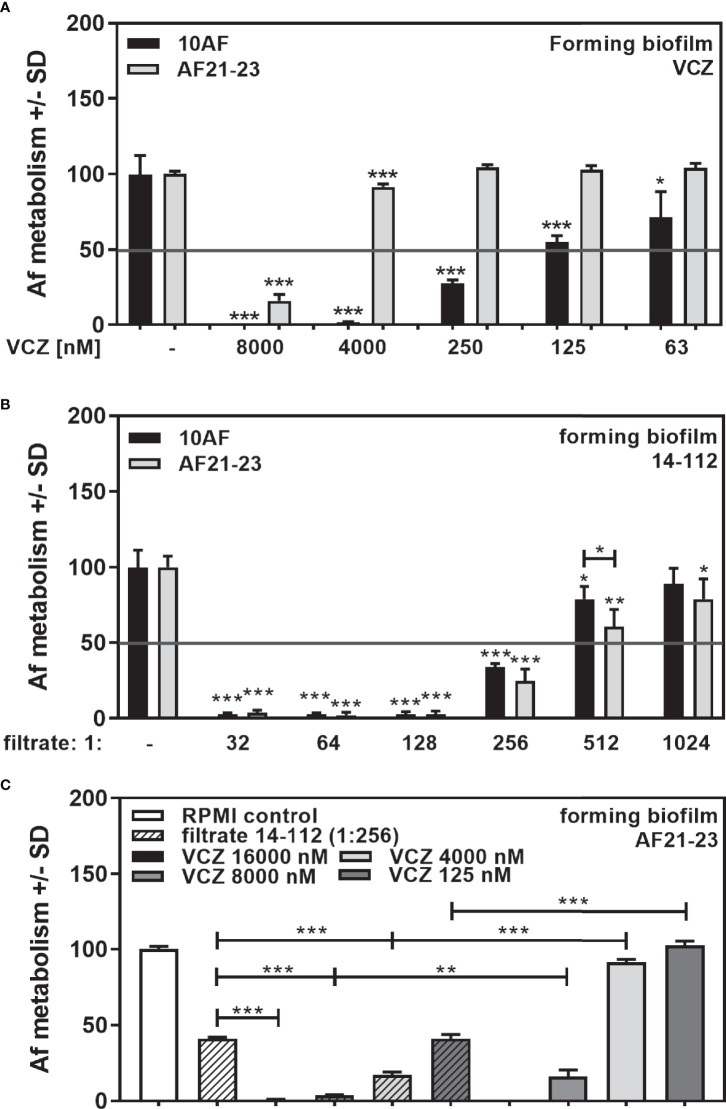
Anti-fungal effects of the combination of *P. aeruginosa* clinical isolate filtrate at 1:256 dilution with VCZ on a VCZ-resistant fungus. VCZ-susceptible (10AF) or VCZ-resistant (AF21-23) *A. fumigatus* were challenged with VCZ [**(A)** 63, 125, 250, 4,000 or 8,000 nM], *Pseudomonas* filtrate [**(B)** isolate 14-112 at 1:32 to 1:1,024 dilution], or **(C)** a combination of VCZ (125. 4,000, 8,000, or 16,000 nM) with 14-112 filtrate (1:256). Comparisons without brackets: untreated fungus vs all treated samples of the same groups. Other comparisons as indicated by the ends of the brackets. Statistical analysis: Unpaired t-test: one, two or three asterisks = p ≤0.05, p ≤0.01 or p ≤0.001, respectively.

As an extreme example of poor VCZ anti-fungal activity we used a clinical *Aspergillus* isolate resistant to VCZ concentrations. We studied concentrations that produced an IC50 in 10AF (**Figure 10A**), and in another wildtype laboratory reference strain, AF13073 (Sass et al., 2021). The VCZ-resistant strain AF21-23 showed an IC50 of 4–8 µM, which is about 50× higher than the IC50 of a susceptible strain (**Figure 10A**). Susceptibility towards *Pseudomonas* anti-fungal activity was similar for the VCZ-susceptible and the VCZ-resistant *Aspergillus* strain (**Figure 10B**). Combination with *Pseudomonas* filtrate synergistically increased VCZ anti-fungal activity even for the resistant fungus (**Figure 10C**).

## Discussion

Co-infection with *A. fumigatus* and *P. aeruginosa* has been described to result in more severe outcome than mono-infection (Amin et al., 2010; Reece et al., 2017), possibly as a result of inflammatory signals caused by intermicrobial competition. On the other hand, numerous *P. aeruginosa* molecules, such as pyoverdine (Sass et al., 2017), phenazines, e.g., pyocyanin (Kerr et al., 1999; Sass et al., 2020), or di-rhamnolipids (Briard et al., 2017) have been shown to interfere with fungal metabolism or growth. The major siderophore pyoverdine was found to be the primary anti-fungal molecule under iron-limited conditions (Sass et al., 2017), whereas phenazine anti-fungal activity was triggered under non-limiting iron conditions (Chatterjee et al., 2020; Sass et al., 2020).

In a recent study, using *P. aeruginosa* laboratory strains PA14 and PAO1, we found synergistic anti-fungal activity of bacterial filtrates and VCZ, independently of the *A. fumigatus* strain used (Sass et al., 2021). The study showed that mediators of synergy encompass pyoverdine and pyocyanin, but also pyochelin, and suggested that the support of VCZ anti-fungal activity by soluble bacterial factors should be taken into consideration when treating *Aspergillus*-*Pseudomonas* co-infections. Such co-infections are not uncommon in persons with CF and other immunocompromised patients (King et al., 2016; O'Brien and Fothergill, 2017). It has been shown that in persons with CF two major *P. aeruginosa* phenotypes exist, mucoid, and non-mucoid CF isolates, of which the mucoid phenotype is associated with lower anti-fungal activity, compared to the non-mucoid phenotype (Ferreira et al., 2015; Nazik et al., 2020). The present study supports the finding of mucoid *P. aeruginosa* isolates from CF patients having less anti-fungal activity than non-mucoid CF isolates by showing that also their interaction with VCZ is weaker. Nevertheless, it is important to stress, all clinical isolates in the present study showed synergistic anti-fungal activity with VCZ, whether used at their individual IC50s, or at a dilution of 1:256, as previously used for testing interaction of VCZ with laboratory isolates (Sass et al., 2021). Synergy was observed over a wide range of filtrate, as well as VCZ, dilutions, increasing the likelihood of such interactions also taking place in the body. Many experiments (not shown) studying VCZ concentrations flanking the VCZ IC50 on both sides (example in **Figure 2**) also affirm the synergy illustrated in this paper. The exceptions were when VCZ or *Pseudomonas* filtrate concentrations, higher than the individual IC50 on their own, already had very strong effects on fungal metabolism. In such cases, synergy could not be calculated using the BLISS Independence Model. Animal studies now have to confirm if our *in vitro* observations of synergy reflect bacterial factor–drug interactions in the body. If stronger VCZ effects are observed during *Aspergillus*–*Pseudomonas* co-infections *in vivo* it might imply the possibility of reducing VCZ therapeutic doses, reducing side effects. On the other hand, treating *Pseudomonas* infection during co-infection with *Aspergillus* might result in the necessity for augmenting VCZ dosing. It is becoming more common to isolate VCZ-resistant *A. fumigatus* strains from CF patients (Hamprecht et al., 2018). Therefore, the observed synergistic effect with the mutant TR34/L98H pan-azole resistant isolate highlights the potential importance of these findings in future therapeutic options. Future studies of interest would include exploring the interaction of VCZ with *A. fumigatus* strains of increased drug vulnerability, such as variants that lack wall galactosaminogalactan.

In general, anti-fungal and synergistic activity of clinical isolates is weaker than that of laboratory isolates, although pyoverdine production is comparable (compare **Table 2** to **Figure 6D** and **Supplemental Figure 1**). In our previous study using laboratory isolates of *Pseudomonas* we identified three bacterial factors that interacted synergistically with VCZ, i.e., pyoverdine, pyocyanin, and pyochelin (Sass et al., 2021). In the present study we encountered four isolates with markedly deficient pyoverdine production (isolates 14-82, 14-89, 14-116, and 14-119) for which we observed similar growth to pyoverdine-positive isolates, measured by OD610. With the loss of pyoverdine production these isolates lost most of their anti-fungal activity, which could be expected, as pyoverdine is a major anti-fungal factor under iron-limiting conditions (Sass et al., 2017). Although pyoverdine seemed to be a major supporter of VCZ anti-fungal activity (Sass et al., 2021), all 4 isolates showed strong synergy with VCZ when used at high concentrations. When used at low concentrations two of the 4 isolates still acted synergistically with VCZ, which suggests the presence of high amounts of anti-fungal factors other than pyoverdine in bacterial filtrates that support VCZ anti-fungal activity. Under the experimental conditions used here (iron-limited medium) it is unlikely that these molecules are phenazines, such as pyocyanin, as pyocyanin induction requires elevated amounts of iron in growth medium (Sass et al., 2020). We also observed that under non-limiting iron conditions very few of our clinical isolates produced pyoyanin, and of the four pyoverdine-negative isolates only 14-119 was able. Synergy of filtrates produced in the presence of iron with VCZ was reduced, compared to filtrates produced under iron-limiting conditions. The third previously identified molecule that supports VCZ anti-fungal activity is pyochelin (Sass et al., 2021). We here could show that rhamnolipids as well are VCZ-synergistic *Pseudomonas* molecules. There are other anti-fungal *Pseudomonas* molecules known that are present in bacterial filtrates, such as 3,4-dihydroxy-2-heptylquinoline (PQS, Nazik et al., 2020), or 4-hydroxy-2-heptylquinoline (HHQ, Nazik et al., 2021), which might add to synergy with VCZ.

Further preliminary data show that harsh heating of pyoverdine negative isolates (95°C for 30 min) significantly reduced their synergistic anti-fungal activity with VCZ (p ≤0.01 for one isolate, p ≤0.001 for 3 isolates). These data indicate that besides the predominant synergistic factor pyoverdine there are other heat-sensitive and heat-stable *Pseudomonas* molecules present that synergistically interact with VCZ against *Aspergillus* forming biofilm metabolism. The repertoire of an individual *Pseudomonas* isolate of produced molecules varies, and it has to be seen if there are common variations within clinical isolate phenotype groups, e.g., mucoid or non-mucoid.

In summary, these data show that clinical *P. aeruginosa* isolates, at varying degrees, synergistically interact with VCZ, and that pyoverdine is not the only molecule responsible.

## Data Availability Statement

The raw data supporting the conclusions of this article will be made available by the authors, without undue reservation.

## Author Contributions

Conceptualization, DAS. Data curation, DAS, GS, PS, and JJM. Formal analysis, DAS, GS, PS, and JJM. Funding acquisition, DAS. Investigation, DAS, GS, PS, and JJM. Methodology, DAS, GS, PS, and JJM. Project administration, DAS and GS. Resources, DAS and RS. Software, GS. Supervision, DAS and GS. Validation, DAS, GS, PS, and JJM. Visualization, DAS, GS, PS, and JJM. Writing—original draft, GS. Writing—review & editing, DAS, GS, PS, JJM and RS. All authors listed have made a substantial, direct, and intellectual contribution to the work and approved it for publication.

## Funding

These studies were funded by ongoing support by the Foundation for Research in Infectious Diseases (FRID, grant 8201). The funder had no role in study design, data collection and interpretation, or the decision to submit the work for publication.

## Conflict of Interest

The authors declare that the research was conducted in the absence of any commercial or financial relationships that could be construed as a potential conflict of interest.

## Publisher’s Note

All claims expressed in this article are solely those of the authors and do not necessarily represent those of their affiliated organizations, or those of the publisher, the editors and the reviewers. Any product that may be evaluated in this article, or claim that may be made by its manufacturer, is not guaranteed or endorsed by the publisher.

## Abbreviations

CF, Cystic Fibrosis; Pa, *Pseudomonas aeruginosa*; VCZ, Voriconazole; Af, *Aspergillus fumigatus*; S, Synergy; I, Independence; A, Antagonism; Y_a_, inhibition of fungal metabolism by drug A; Y_b_, inhibition of fungal metabolism by drug B; Y°_ab_, observed combined antifungal effect; ${\text{Y}}_{\text{ab}}^{\text{p}}$ , predicted combined antifungal effect; inhibitory concentration of 50% (IC50).
