# Supplementary figures and images for: Synergy Between Pseudomonas aeruginosa Filtrates And Voriconazole Against Aspergillus fumigatus Biofilm Is Less for Mucoid Isolates From Persons With Cystic Fibrosis
Source: Front Cell Infect Microbiol. 2022 Apr 14;12:817315. doi: 10.3389/fcimb.2022.817315 (PMC9047052; doi:10.3389/fcimb.2022.817315)

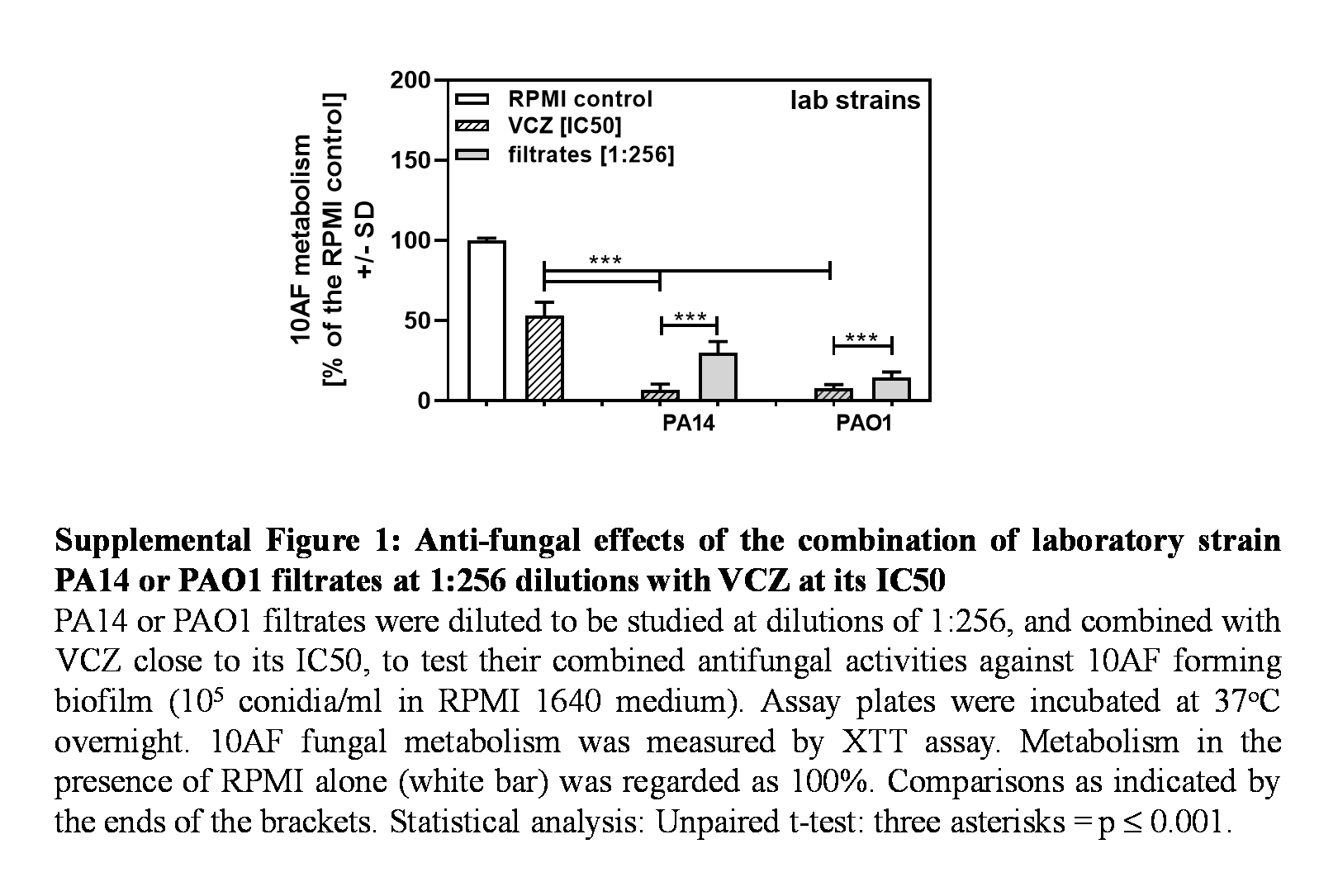

Supplement: Supplementary file 1 [file Image_1.tif]
